# “WE WILL SURVIVE … BUT I JUST WANT MORE FOR US”: DISSEMINATING PHOTOVOICE FINDINGS TO ADVANCE FOOD POLICY AND EQUITY

**DOI:** 10.1093/geroni/igac059.1496

**Published:** 2022-12-20

**Authors:** Anika Hines, Rebecca Brody, Zehui Zhou, Kennedy McDaniel, Chiazam Omenyi, Edgar Miller, III, Lisa Cooper, Deidra Crews

**Affiliations:** Virginia Commonwealth University School of Medicine, Richmond, Virginia, United States; Williams College, Williamstown, Massachusetts, United States; Johns Hopkins Center for Health Equity, Baltimore, Maryland, United States; Johns Hopkins School of Nursing, Baltimore, Maryland, United States; Johns Hopkins Center for Health Equity, Baltimore, Maryland, United States; Johns Hopkins School of Medicine, Baltimore, Maryland, United States; Johns Hopkins School of Medicine, Baltimore, Maryland, United States; Johns Hopkins University, Baltimore, Maryland, United States

## Abstract

Poor food environments and disproportionate food insecurity among African Americans (AAs) represent determinants of chronic disease disparities by race/ethnicity. We used PhotoVoice to explore the food environment in Baltimore, MD, through the perspectives of Black residents with hypertension. Findings illuminated structural racism and classism as root causes of “food desert” conditions. Respondents provided critical analysis of existing food access barriers, including problematic policies, and offered asset-based solutions. Using an exhibition of participants’ photographs to set the tone of the discussion, we will convene a stakeholder meeting including study participants, community advisory board members, local and state government agencies, civic organizations, community farmers, agricultural organizations, non-profit food providers, churches, local restauranteurs, convenience store owners, insurance payers, and transportation providers. Discussion will center on three areas of action informed by findings: systems level issues; individual activation, agency, and advocacy; and community-based solutions. Meeting proceedings will be disseminated through academic and lay publications.

